# Supplementary material for: Ammonia dimer: extremely fluxional but still hydrogen bonded
Source: Nat Commun. 2022 Mar 18;13:1470. doi: 10.1038/s41467-022-28862-z (PMC8933541; doi:10.1038/s41467-022-28862-z)
Supplement: Supplementary file 1 — Supplementary Information [file 41467_2022_28862_MOESM1_ESM.pdf]

Supplementary Information:

Ammonia dimer: extremely fluxional but still hydrogen bonded

A. Jing *et al.*

SUPPLEMENTARY METHODS

Electronic structure calculations

The *ab initio* electronic structure calculations on which our potential energy surface (PES) is based are described in the main text and in Methods. Here we provide more details. The CCSD(T)/FC calculations were performed using the Dunning augmented valence bases: aug-cc-pVXZ [1, 2] X = Q and 5, while the CCSD(T)/AE calculations were performed using an augmented core-valence basis: aug-cc-pCVXZ [3], with X = T. We will abbreviate aug-cc-pVXZ as aXZ, aug-cc-pCVXZ as aCXZ, and so on. The (3s2p1d) set of midbond functions from Ref. 4 was used with the aXZ and aCXZ basis sets. The CCSD(T) calculations were performed using the MOLPRO 2010.1 [5] program. The CCSDT(Q) calculations were performed using the MRCC package [6] and the jun-cc-pVDZ basis (jDZ) from Ref. 7. We used an exponential formula for the Hartree-Fock (HF) energy with a fixed exponent equal to 1.63 recommended in Ref. 8 and an X^{-3} formula for the correlation energy to obtain CBS limits.

The geometries of the lowest three stationary points (SPs) are listed in Supplementary Table 1 and plotted in Fig. 1 of the main text. Also the experimental structure [9–11] is shown Fig. 1. These SPs all have a symmetry plane, so their geometries are fully characterized by the intermonomer separation and the two angles given in the table. Supplementary Figure 1 visualizes the motion along the interconversion path between SP#2 and SP#0.

Supplementary Table 1

Geometries of the lowest three stationary points (distances in Å, angles in degrees). Also the interaction energies (in kcal/mol) and barriers (in cm^{-1}) are listed, both from the fit and from *ab initio* calculations at the fit geometries. All three configurations have a plane of symmetry. $\angle$NNH$_\mathrm{A}$ and $\angle$NNH$_\mathrm{B}$ are the angles that the in-plane hydrogens make with the N-N axis for monomers A and B, respectively.

	E_int(fit)/barrier	E_int(*ab initio*)/barrier	R	r_NN	$\angle$NNH$_\mathrm{A}$	$\angle$NNH$_\mathrm{B}$
SP#0	–3.24898/0.00	–3.25000/0.00	3.273	3.222	20.8	70.3
SP#1	–3.23399/5.24	–3.23418/5.53	3.218	3.167	42.6	42.6
SP#2	–3.18594/22.1	–3.18463/22.9	3.311	3.259	12.8	139.3

Supplementary Figure 1

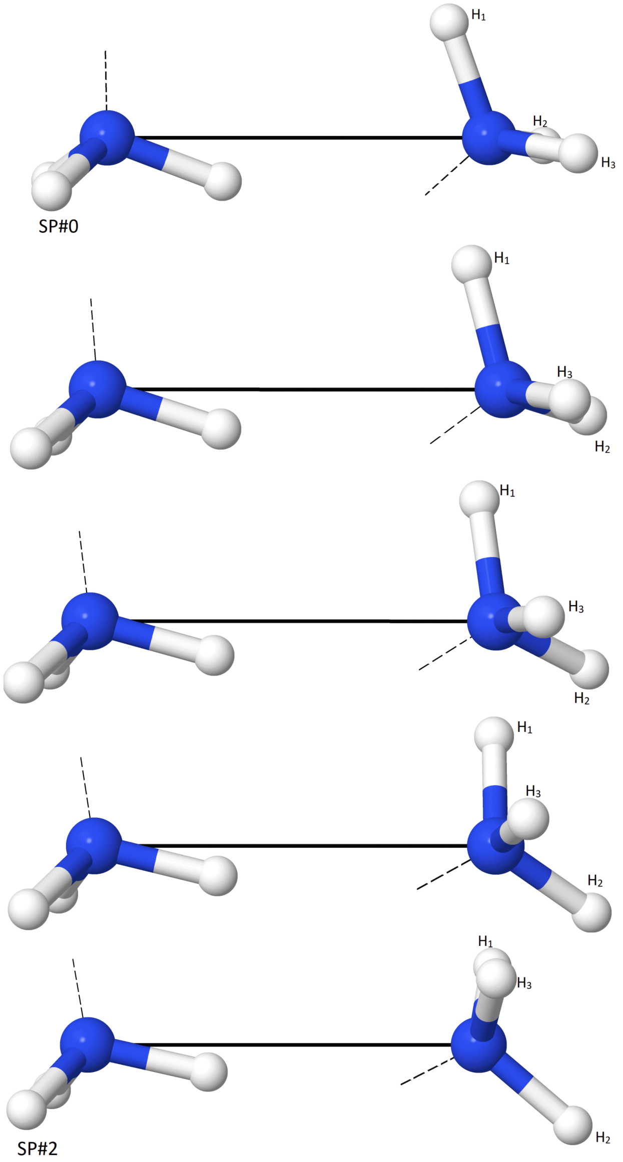

Interconversion path between SP#2 and SP#0.

Form of fit function

The fit was in the form of a sum of isotropic functions of intermolecular site-site distances, plus a polarization term

$$V = \sum_{a \in A} \sum_{b \in B} u_{ab} + V_{\mathrm{ind}}(A, B). \qquad \text{(Supplementary Equation 1)}$$

All atoms are sites and the positions of off-atomic sites were determined as described below.

Each site-site function has the form

$$u_{ab} = A_{\rm u}\left[1 + a_1^{ab} r_{ab} + a_2^{ab}(r_{ab})^2 + a_3^{ab}(r_{ab})^3\right] e^{\alpha^{ab}} e^{-\beta^{ab} r_{ab}} + \frac{A_{12}^{ab}}{(r_{ab})^{12}} + f_1(\delta_1^{ab}, r_{ab})\frac{q_a q_b}{r_{ab}} + \sum_{n=6,8,10} f_n(\delta_n^{ab}, r_{ab})\frac{C_n^{ab}}{(r_{ab})^n},$$

(Supplementary Equation 2)

where r_{ab} are the distances between sites, q_x are partial charges, C_n^{ab} are van der Waals coefficients, f_n are Tang-Toennies [12] damping functions with damping parameters δ_n^{ab}, and $A_{\rm u}$ is a constant equal to 1 kcal/mol. The coefficients A_{12}^{ab} are constrained to be positive and large, so that the term $A_{12}^{ab}/(r_{ab})^{12}$ ensures a repulsive behavior at very close range. Site-site functions u_{ab} involving atoms, numbered as sites 1-4, were of the form specified by Supplementary Equation 2, i.e., they include all the terms. The functions for the off-atomic sites did not include the $C_n^{ab}/(r_{ab})^n$ terms and none of those sites was "polarizable". The functions involving sites number 5-15 included only the exponential, $1/(r_{ab})^{12}$, and electrostatic terms, those involving sites 16-18 included only the exponential and $1/(r_{ab})^{12}$ terms. All details of the fit not specified here were set to the defaults of autoPES [13]. The repulsive wall height was set to at least 50 kcal/mol in scanning for holes in the PES [13]. The polarization term $V_{\rm ind}$ describes interactions due to a set of induced point-dipoles $\boldsymbol{\mu}_c^{\rm ind}$ placed on each atomic site of a monomer

$$V_{\rm ind} = -\frac{1}{2}\sum_{a\in A} \boldsymbol{\mu}_a^{\rm ind}\cdot \boldsymbol{E}_a - \frac{1}{2}\sum_{b\in B} \boldsymbol{\mu}_b^{\rm ind}\cdot \boldsymbol{E}_b, \quad \text{(Supplementary Equation 3)}$$

$$\boldsymbol{\mu}_a^{\rm ind} = \alpha_a\left[\boldsymbol{E}_a + \sum_{b\in B} \boldsymbol{T}_{ab}\boldsymbol{\mu}_b^{\rm ind}\right] \quad \text{(Supplementary Equation 4)}$$

and similarly for $\boldsymbol{\mu}_b^{\rm ind}$, where α_a is an isotropic polarizability of atom a, $\boldsymbol{E}_a$ is the damped electric field at the position of atom a due to all (permanent) point charges of monomer B,

$$\boldsymbol{E}_a = \sum_{b\in B} f_1(\delta_1^{ab}, r_{ab})\frac{q_b \boldsymbol{r}_{ab}}{r_{ab}^3}, \quad \text{(Supplementary Equation 5)}$$

and $\boldsymbol{T}_{ab}$ is the damped dipole-dipole interaction tensor,

$$\boldsymbol{T}_{ab} = f_3(\delta_{\rm p}^{ab}, r_{ab})\left[3\frac{\boldsymbol{r}_{ab}\otimes\boldsymbol{r}_{ab}}{r_{ab}^5} - \frac{\mathbf{1}}{r_{ab}^3}\right], \quad \text{(Supplementary Equation 6)}$$

where δ_1^{ab} and $\delta_{\rm p}^{ab}$ are damping parameters. The parameters $a_i^{ab}, A_{12}^{ab}, \alpha^{ab}, \beta^{ab}, \delta_i^{ab}$, and $\delta_{\rm p}^{ab}$ were found by a least-squares fit to the close-range set of training grid points. A penalty function [13] was used to constrain the parameters A_{12}^{ab}. The asymptotic parameters q_x, C_n^{ab}, and

α_x were determined from *ab initio* computed properties of monomers at the level of theory consistent with SAPT(DFT) [13] and using the a5Z basis set. These parameters were kept fixed in fitting the remaining parameters. The positions of sites 5-15 (5 symmetry-distinct sites) were optimized together with q_x's by fitting the *ab initio* computed COM multipole moments of ammonia molecule. The positions of sites 16-18 (one symmetry-distinct site) were fitted together with other parameters during the close-range optimization [13, 14].

Calculation of the vibration-rotation-tunneling states

As explained in Methods, the vibration-rotation-tunneling (VRT) states are calculated with a pseudo-spectral method, implemented in a computer program developed by Leforestier [15, 16]. This method applies a split basis: an analytic basis consisting of coupled symmetric rotor functions for the angular coordinates, multiplied with a set of radial sine-type basis functions, and a corresponding 6D direct-product grid basis in the six internal coordinates $R, \beta_A, \gamma_A, \alpha_B, \beta_B, \gamma_B$. The number of radial basis functions was 38, the radial grid consisted of 41 equidistant points in the range from 2.4 to 6.5 Å. We checked these parameters by making also calculations with 50 basis functions and 56 grid points ranging from 2.4 to 8.2 Å. The energy levels differed by less than 0.0003 cm^{-1}. The convergence of the angular basis was checked by making calculations with the maximum monomer rotational quantum numbers j_A, j_B of 13 in the basis (while these values were 12 for all results reported). The levels of NH_3-NH_3 were lower by about 0.02 cm^{-1} with the larger basis, those of ND_3-ND_3 by about 0.1 cm^{-1}. The energy level differences that we compare with experimental values were smaller for the larger basis by less than 0.01 cm^{-1} for NH_3-NH_3 and less than 0.08 cm^{-1} for ND_3-ND_3. The slower convergence for ND_3-ND_3 may be expected, because the rotational constants of ND_3 are only about half of those for NH_3. Hence, the hindered internal rotor states of ND_3-ND_3 are more localized and thus more difficult to converge in a free-rotor basis. We used experimental values for the monomer rotational constants: $A = B = 9.9402$ cm^{-1} and $C = 6.3044$ cm^{-1} for NH_3, $A = B = 5.1432$ cm^{-1} and $C = 3.1015$ cm^{-1} for ND_3. The atomic masses are: $m_{\text{N}} = 14.0030740052$ u, $m_{\text{H}} = 1.0078250321$ u, $m_{\text{D}} = 2.014101778$ u.

SUPPLEMENTARY DISCUSSION

Supplementary Figure 2

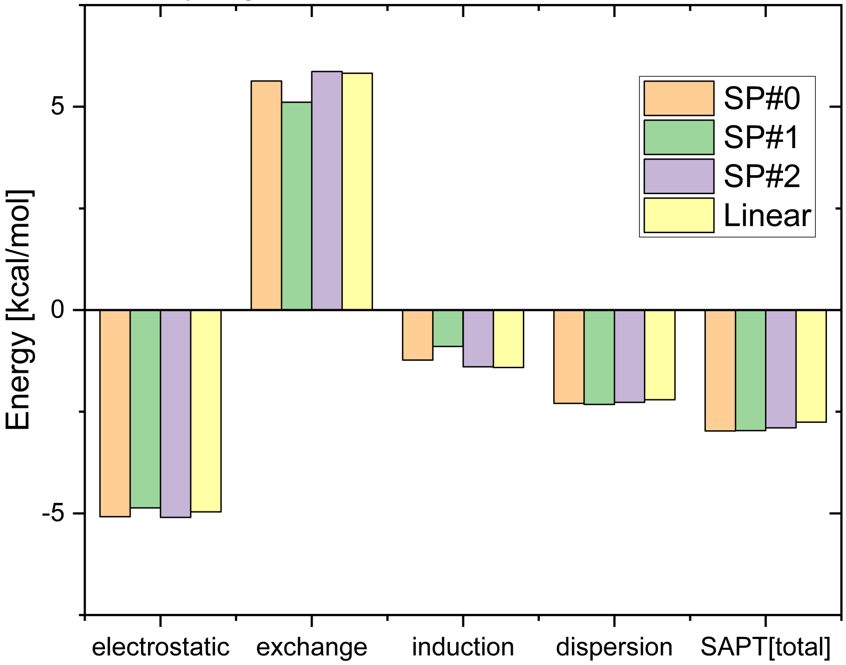

Comparison of SAPT components at the SP#0, SP#1, SP#2, and staggered linear hydrogen bond configurations.

SAPT components

SAPT components on the path from the linear to SP#0 to SP#1 structures are shown in Fig. 3 of the main text. The staggered linear hydrogen bond configuration is depicted in Fig. 1 of the main manuscript. This configuration is defined by the linearity of hydrogen bond, i.e., the N-H-N atoms (with H in the hydrogen bond) form a straight line, the presence of a symmetry plane C_s, and the staggered position of the remaining hydrogens. With the Euler angles determined by these conditions (different for each value of R), the radial minimum was found with an interaction energy of -3.040 kcal/mol at $R = 3.3624$ Å. The Euler angles at this configuration are: $\beta_A = 111°$, $\gamma_A = 60°$, $\alpha_B = 0°$, $\beta_B = 178°$, and $\gamma_B = 0°$ (note that the N atoms are in general not on $\boldsymbol{R}$). Interestingly, the eclipsed linear dimer has an almost identical interaction energy on our PES, -3.034 kcal/mol, and a separation $R = 3.3634$ Å. The PES energy of SP#1 (SP#2) is 5 (22) cm^{-1}. The corresponding energies at the SAPT level used here are 3 (26) cm^{-1}. The accuracy of SAPT results could be improved, but this was not needed since we use SAPT only for interpretative purposes. The paths in the configuration space shown in Fig. 3 of the main text were determined by following the steepest descent on the PES, with numerically computed gradient, starting

from either the linear configuration or from SP#1 and ending at the global minimum. Then, at each point on the path, SAPT calculations were performed. For the SP#1 to SP#0 path, the C_s symmetry was imposed, so only R and the two β angles we varied. For the linear to SP#0 path, also the angle γ_B was varied. The curves in Fig. 3 of the main text are less smooth on the path linear–SP#0 than on SP#0–SP#1 since the change of geometry is much more substantial in the former case as we have to go from a staggered configuration to an eclipsed one.

Supplementary Figure 2 presents the values of the components at the three characteristic points included in Fig. 3 of the main text and in addition at SP#2. It shows the actual values, rather than values relative to SP#0. One striking observation is that the values of essentially all the components are very close to each other for all four geometries, although these geometries are rather distinct. For both the electrostatic and dispersion components, the largest difference relative to the SP#0 values is only 4%. While the dispersion energy is known to be fairly isotropic, this behaviour is not expected of the electrostatic energy. The anisotropy of the exchange energy ranges between 3% and 9%, and is the major factor defining the shape of the PES near the global minimum, as discussed in the main text. Somewhat surprisingly, the most anisotropic component is the induction energy, with relative differences from 13% to 27%. However, since induction is relatively small in magnitude compared to other components, its variation in absolute terms is in line with other contributions.

Supplementary Figure 3

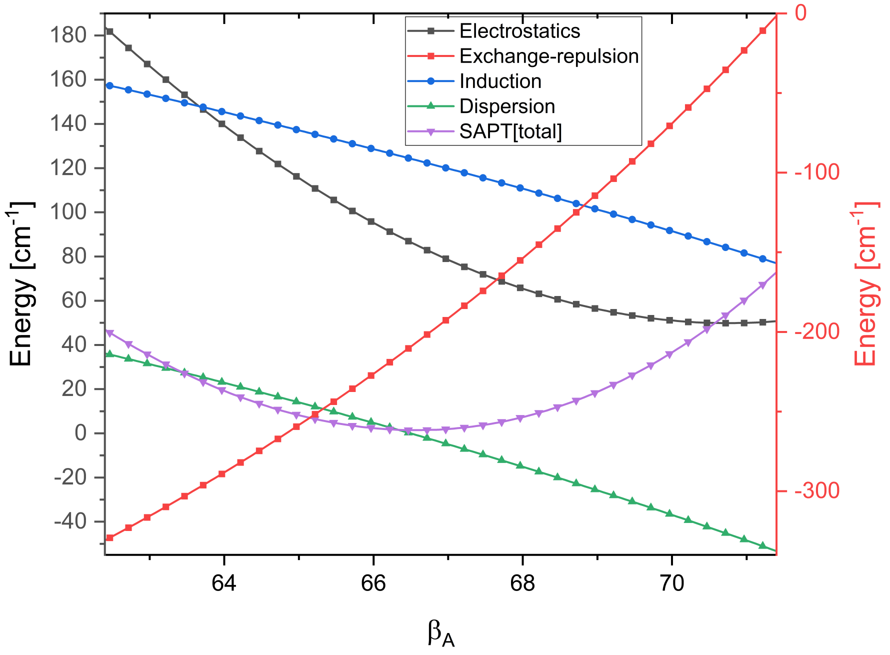

SAPT components for a path through SP#1 perpendicular to the direction of the potential valley in Fig. 2(a) of the main text: $180 - \beta_B = \beta_A$ (in degrees). In contrast to Fig. 2(a), R is held constant at its SP#1 value. All energies are relative to their values at SP#0.

Supplementary Figure 3 shows the behaviour of SAPT components when the dimer moves across the valley in Fig. 2(a) of the main text, passing through SP#1 at $\beta_A = 67.2°$. The increase of β_A corresponds to the inner hydrogens getting closer to each other. This results in the exchange energy becoming significantly more repulsive, which is the main reason for the steepness of the wall since it overcomes the effects of the remaining components that all become more attractive. On the other hand, when β_A decreases the exchange component becomes less repulsive, although at a somewhat smaller rate than for the increase of this angle. All the attractive components become sufficiently less attractive to form the other wall of the valley. In particular, the change of the electrostatic component is large. This change is to a large extent driven by penetration (overlap) effects that make it more attractive than the asymptotic value. Thus, as β_A decreases, the penetration effects become smaller and the electrostatic energy becomes less attractive. Since also the exchange effects are approximately proportional to the density overlap, this overlap is the main factor shaping the PES in the region of the minima.

Vibration-rotation-tunneling states

The numerical VRT levels are presented in Supplementary Table 2 in such a way that they can be directly compared with the levels deduced from experimental high-resolution spectra [17]. That is, all energies are listed relative to the $J = 0$ ground state energies E_0 of the states with A_1, E_1, and G symmetry. These E_0 values are given with respect to the same limit of $j = 0$ monomers at infinite separation. Notice that the E_0 states are not always the lowest states of a given symmetry. For $J = 0$, the E_3 state lies slightly below E_1, while the E_1 state for $J = 1$ lies ≈ 2 cm^{-1} below the E_1 state for $J = 0$. The result that the lowest state occurs for $J = 1$ is not uncommon for van der Waals complexes with NH_3 constituents. It is related to the fact that *para*NH_3 with ground state $j_k = 1_1$ more easily finds the most favorable orientation in the complex than *ortho*NH_3 with ground state $j_k = 0_0$ and that this monomer angular momentum is partly conserved in the complex. Nevertheless, we used the $J = 0$ A_1, E_1, and G states to determine the dissociation energies. As explained in the main text, the dissociation energies D_0 are the negatives of the E_0's plus the $j_k = 1_1$ isolated monomer energy for G symmetry and twice this value for E symmetry.

Supplementary Figure 4

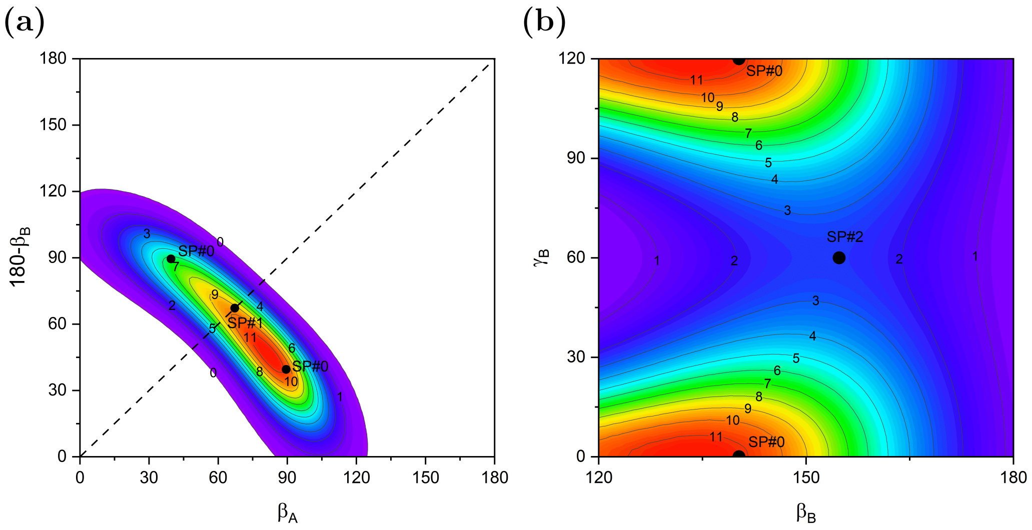

ND_3-ND_3 ground state wave function of G symmetry, absolute value squared in bohr^{-3}. Panels (a) and (b) show this wave function in the same regions where the NH_3-NH_3 wave function is shown in Fig. 5 of the main text. Note that in panel (a) the ND_3-ND_3 wave function has a larger density in the region of the potential minima SP#0, but that the maximum is closer to the cyclic SP#1 structure than for NH_3-NH_3. Panel (b) shows that the density at the SP#2 barrier is relatively smaller than for NH_3-NH_3, but still considerable.

Supplementary Table 2

Calculated energy levels (in cm^{-1}) of NH_3-NH_3 relative to the $J = 0$ ground state levels of A_1, E_1, and G symmetry compared with the experimental levels from high-resolution spectra [17].

Ground state and dissociation energies		
Symmetry	E_0	D_0
A_1	–690.9208	690.92
E_1	–681.6806	714.17
G	–687.5821	703.83

Relative energies		
Symmetry	Theory	Experiment
	$J = 0$	
A_1	0.0	0.0
A_4	17.2877	16.1211
E_1	0.0	0.0
E_2	20.9642	19.3573
E_3	–0.0038	–0.0058
E_4	20.8938	19.2332
G	0.0	0.0
G	21.5557	20.4867
RMSE	1.25	
	$J = \|K\| = 1$	
A_1/A_2	7.3065/7.3068	7.3652/7.3655
A_3/A_4	25.9404/25.9388	24.6542/24.6526
E_1	–1.7871	–2.2998
E_1	9.5150	9.9379
E_2	14.6211	12.8326
E_2	37.0500	35.4641
E_3/E_4	1.7235/1.7330	1.2651/1.2780
E_3/E_4	26.7036/26.7039	25.6346/25.6349
G	4.0402	4.0873
G	7.8322	8.0258
G	21.5018	20.3123
G	33.6756	32.9436
RMSE	0.94	
	$J = \|K\| = 2$	
E_2	25.0339	23.3160
G	18.3969	18.2793
G	24.3799	
G	40.2641	39.5574
RMSE	1.08	

Supplementary References

[1] Dunning, Jr., T. H. Gaussian-basis sets for use in correlated molecular calculations. 1. The atoms boron through neon and hydrogen. *J. Chem. Phys.* **90**, 1007–1023 (1989).

[2] Kendall, R. A., Dunning, Jr., T. H. & Harrison, R. J. Electron-affinities of the 1st-row atoms revisited - systematic basis-sets and wave-functions. *J. Chem. Phys.* **96**, 6796–6806 (1992).

[3] Woon, D. E. & Dunning Jr., T. H. Gaussian basis sets for use in correlated molecular calculations. V. Core-valence basis sets for boron through neon. *J. Chem. Phys.* **103**, 4572–4585 (1995).

[4] Mas, E. M., Szalewicz, K., Bukowski, R. & Jeziorski, B. Pair potential for water from symmetry-adapted perturbation theory. *J. Chem. Phys.* **107**, 4207–4218 (1997).

[5] Werner, H.-J. *et al.* MOLPRO, version 2010.1, a package of ab initio programs (2010). See http://www.molpro.net.

[6] Kállay, M. *et al.* MRCC, a quantum chemical program suite. *URL: http://www.mrcc.hu, accessed August 26th* (2016).

[7] Papajak, E. & Truhlar, D. G. Convergent partially augmented basis sets for post-Hartree-Fock calculations of molecular properties and reaction barrier heights. *J. Chem. Theory Comput.* **7**, 10–18 (2011).

[8] A. Halkier, W. Klopper, T. Helgaker, P. Jørgensen, and P. R. Taylor. Basis set convergence of the interaction energy of hydrogen-bonded complexes. *J. Chem. Phys.* **111**, 9157–9167 (1999).

[9] Nelson Jr., D. D., Fraser, G. T. & Klemperer, W. Ammonia dimer: A surprising structure. *J. Chem. Phys.* **83**, 6201–6208 (1985).

[10] Nelson Jr., D. D., Klemperer, W., Fraser, G. T., Lovas, F. J. & Suenram, R. Ammonia dimer: Further structural studies. *J. Chem. Phys.* **87**, 6364–6372 (1987).

[11] Nelson Jr., D. D., Fraser, G. T. & Klemperer, W. Does ammonia hydrogen bond? *Science* **238**, 1670–1674 (1987).

[12] Tang, K. T. & Toennies, J. P. An improved simple-model for the van der Waals potential based on universal damping functions for the dispersion coefficients. *J. Chem. Phys.* **80**, 3726–3741 (1984).

[13] Metz, M. P., Piszczatowski, K. & Szalewicz, K. Automatic generation of intermolecular potential energy surfaces. *J. Chem. Theory Comput.* **12**, 5895–5919 (2016).

[14] Metz, M. P. & Szalewicz, K. Automatic generation of flexible-monomer intermolecular poten-

tial energy surfaces. *J. Chem. Theory Comput.* **16**, 2317–2339 (2020).

[15] Leforestier, C. Grid method for the Wigner functions. Application to the van der Waals system Ar-H_2O. *J. Chem. Phys.* **101**, 7357–7363 (1994).

[16] Leforestier, C., Braly, L. B., Liu, K., Elrod, M. J. & Saykally, R. J. Fully coupled six-dimensional calculations of the water dimer vibration-rotation-tunneling states with a split Wigner pseudo spectral approach. *J. Chem. Phys.* **106**, 8527–8644 (1997).

[17] Loeser, J. G. *et al.* Multidimensional hydrogen tunneling dynamics in the ground vibrational state of the ammonia dimer. *J. Chem. Phys.* **97**, 4727–4749 (1992).
