# Supplementary material for: Ammonia dimer: extremely fluxional but still hydrogen bonded
Source: Nat Commun. 2022 Mar 18;13:1470. doi: 10.1038/s41467-022-28862-z (PMC8933541; doi:10.1038/s41467-022-28862-z)
Supplement: Supplementary file 3 — Description of Additional Supplementary Files [file 41467_2022_28862_MOESM3_ESM.docx]

**Description of Additional Supplementary Files**

**File Name:** Supplementary Data 1

**Description:** The file Supplementary_Data_1.zip contains a FORTRAN program computing the fitted potential, parameters of the fit, instructions on using the program, as well as coordinates and energies of all computed data points.
